# Envisioning Home Health as a Critical Component of Learning Health Systems to Improve Evidence-Based Care in Heart Failure

**DOI:** 10.1016/j.jacadv.2025.101980

**Published:** 2025-07-23

**Authors:** Madeline R. Sterling, Lisa M. Kern, Margaret V. McDonald, Jonathan N. Tobin, Alicia I. Arbaje, Crystal W. Cené, Christine D. Jones, Michael Dicpinigaitis, Michelle Shum, Kathryn H. Bowles

**Affiliations:** a Department of Medicine, Weill Cornell Medicine, New York, New York, USA; b Center for Home Care Policy & Research, VNS Health, New York, New York, USA; c Clinical Directors Network (CDN), New York, New York, USA; d The Rockefeller University Center for Clinical and Translational Science, New York, New York, USA; e Johns Hopkins University, School of Medicine and Bloomberg School of Public Health, Baltimore, Maryland, USA; f UC San Diego (UCSD) Health and UC San Diego School of Medicine, California, USA; g University of Colorado Denver - Anschutz Medical Campus, and Rocky Mountain Regional VA Medical Center, Aurora, Colorado, USA; h University of Pennsylvania School of Nursing, Philadelphia, Pennsylvania, USA

**Keywords:** heart failure, home health agency, home health care, hospital, learning health system

The concept of a “learning health system” was first articulated nearly 20 years ago by the National Academy of Medicine, but it has not yet been fully realized.^1^ The concept of a learning health system grew out of related concepts like evidence-based medicine, which are now widely embraced. A learning health system (LHS) is a health care system that collects data on how it delivers care and then uses that data to continuously improve care delivery. LHSs are, for the most part, based in hospitals and their affiliated outpatient networks.^2^ In this Viewpoint, we contend that LHSs have not yet included home health care (HHC) and propose that including HHC could improve the depth and breadth of data available for continuous quality improvement, particularly for patients with heart failure. However, several barriers exist that need to be addressed before fully integrating HHC into LHSs.

## The status of home health care in learning health systems

The term “home health care” refers to the delivery of care for patients in their homes by a variety of providers, including nurses, physical therapists, occupational therapists, social workers, and home health aides. HHC, which is provided by Medicare certified home health agencies, is often initiated at the time of hospital discharge to facilitate the transition home and assist with avoiding preventable readmission. HHC is particularly common for patients with heart failure, with up to one-third of the 6 million adults with heart failure in the United States referred to HHC.^3^ For patients with heart failure, home care nurses monitor symptoms, perform medication reconciliation, monitor vital signs, while physical therapists address mobility and function, and home health aides provide assistance with personal care, including activities of daily living, reminding patients to take medication, and assisting with doctors’ appointments.^4^ A federally mandated, standardized assessment is completed at admission and discharge and other time points during a HHC episode. This data set, called the Outcomes Assessment Information Data Set, contains over 100 clinical, service, and functional status measures of patients and their outcomes. In addition to a thorough assessment on admission, the HHC workforce is in the home observing and advising patients for hours at a time. As such, they can serve as vital “eyes and ears,” with ability to assess environmental and behavioral conditions not possible to observe in acute or outpatient settings, including lack of compliance with medication or the care plan, weight gain, what foods are available, falls risk factors, or the development of new symptoms, including leg swelling and shortness of breath, before anyone else does. Because patients with heart failure may be vulnerable to rapid decompensation, timely attention to worsening symptoms is extremely useful.

If successful, LHSs use the data generated from patient care to improve outcomes. Over the past 20 years, the development of electronic health records (EHRs) facilitates the reuse of health care data for quality improvement and research. However, outside of acute care, challenges with EHR interoperability (the ability to exchange data seamlessly) impede communication and availability of data. This challenge is common in free-standing (nonintegrated), financially independent (not owned by a health system) home health agencies, due to different brands of EHRs and lack of incentives to collaborate on improvement. Sadly, the rich information about hospitalization and the unique data generated in the home when patients receive HHC do not routinely make their way back to providers and other clinicians in the LHS. If those observations could make it back, then the LHS might be able to use that information to reduce the high rates of readmission that patients with heart failure current face. Currently, 20% to 25% of Medicare beneficiaries, discharged after a heart failure exacerbation, are readmitted within 30 days, with most of those readmissions occurring within the first 15 days postdischarge, and prior efforts have not been successful at reducing this rate.

## Barriers impeding the integration of home health care into learning health systems and ways to overcome them

Integrating HHC into the LHS has the potential to better align the care that patients are receiving, including the data collected and observations made across the care continuum. Indeed, qualitative studies with a variety of stakeholders, ranging from physicians to home health agency leaders and staff, suggest that many of them would like a better-connected system—one in which home health agencies, the hospital, and ambulatory care worked together in a more unified fashion to care for patients.^4^ However, several barriers currently impede this integration.^5^ While some are at the provider level, others are structural in nature.

Barriers at the provider level currently limit the uptake and integration of HHC services; however, several of these could be addressed with targeted interventions. First, increased awareness and partnership from acute- and ambulatory care-based clinicians—particularly general internists, cardiology, and other subspecialists—is needed. Not only can physicians recognize when a patient may benefit from and qualify for HHC and place the referral, but they can also encourage patients to avail themselves of HHC services. So often, and understandably, patients are discharged home with HHC but may decline to participate in physical therapy or nurse visits because they are too tired, sick, or have competing priorities. Physicians can encourage them to do their best at these visits, and involve their caregivers, so that even if they are physically unable, the cognitive aspects of care can be covered. Second, when home health agencies begin the start of care, physicians can provide a “warm handoff” (ie, an electronic or telephone-based summary of the hospitalization and what to look out for in the home) to the agency but also give them ways to keep in touch between visits if they notice that a patient is deteriorating. The quality of information on the referral documents and the discharge summary is critically important to communicate the clinical status of the patient to HHC. This information alerts the next level of care of risk factors and prompts a timely start of care and attention to those who need it most.

At the organizational level, barriers exist particularly with information exchange and communication. Currently, information flows poorly between the hospital, patient, and home. For example, while referrals for patients to receive HHC are initiated in the health system, they can take days to be physically signed, a necessary step to begin the start of care. Additionally, the hospital discharge summary rarely makes it to the home health agency in a timely fashion. This leaves the HHC clinician with insufficient information about the patients’ clinical status during the first in-home assessment, occurring shortly after hospital discharges which are often chaotic and frightening to patients and caregivers, and can be highly detrimental in the case of heart failure. Additionally, when HHC nurses identify issues with medications, or home health aides observe new symptoms in the home, there are insufficient channels to alert physicians and receive their feedback quickly. Our team and others are working to address some of these issues. Pilot studies of hospital-to-home programs which provide home health clinicians and patients tools for expectation setting, clarification of roles, information management, and care transition quality rating are beginning to show promise.^6^ We have also made advances in enabling home health aides to have access to new technology that allows them to text message clinical supervisors in real time to enable faster and more complete communication about their patients’ clinical status.^7^ Despite these steps forward, full data integration and reliable communication pathways remain elusive.

To that end, and to truly achieve a LHS that can better support patients, system-level barriers need to be addressed so that home health agencies can be integrated into the health system, including complete and timely information flow. This would allow for the proper exchange of data, with iterative feedback and quality improvement. If HHC were integrated into the LHSs, data from the agency could stimulate discussion and systematic change to improve care. For example, findings that a significant portion of patients referred to HHC were turning down start of care visits on weekends after being discharged on Friday afternoon might change discharge practices at the hospital. This could lead to better acceptance rates of HHC, while in turn, reducing readmissions. Additionally, reports on errors found when completing medication reconciliation by HHC clinicians could be quickly viewed by physicians in the health system, who could not only learn from the process but also adjust in real-time. While such integration would require initial resources and incentives to collaborate and dismantle existing silos, it could have major return on investment for hospitals, agencies, clinicians, and most importantly, patients.

## Leveraging implementation science to facilitate the integration of home health care into learning health systems

Once established, the LHS with integrated HHC could leverage principles of implementation science to improve health care delivery and outcomes. Implementation science is the scientific study of methods and strategies that facilitate the uptake of evidence-based practice and research into regular use by practitioners and policymakers.^8^ Implementation science is a core component of LHS, since it aims to address and overcome the barriers that slow or halt the uptake of proven health interventions and evidence-based practices and de-implement useless or harmful practices. An going trial, the I-TRANSFER-HF (Improving Transitions of Care For Heart Failure Patients Receiving Home Health Care) study, aims to improve HF care transitions and outcomes through early and intensive HHC nurse visits alongside early outpatient follow-up.^7^ Using a hybrid type 1 design, the study is testing the effectiveness of the I-TRANSFER-HF intervention in a stepped wedge cluster randomized controlled trial and determining the context surrounding its implementation among a diverse sample of hospitals and their referring home health agencies (dyads) across the United States. This, as well as the ongoing I-TRANSFER study (which focuses on HHC for sepsis survivors),^9^ are the first pragmatic trials of HHC which are intentionally bringing hospitals, home health agencies, and ambulatory care practices together to increase the uptake and integration of evidence-based practices for patients who receive HHC. The results have the potential to inform clinical care and health care delivery strategies for these high-risk patient populations. They will also demonstrate how a hospital-HHC LHS works, and its impact on outcomes.

## Conclusions

Ultimately, a LHS that integrates HHC could lead to better patient outcomes. Before such a dramatic change occurs, however, there are many individual steps that health care providers, patients, and health system leaders can undertake now toward achieving this vision. At the clinician level, increased awareness of HHC and its potential benefits to improve care in HF is necessary. This includes educating providers of the value of HHC to increase referrals to HHC when clinically appropriate and encouraging patients to avail themselves of HHC after discharge. Hospitals should support integration and partnerships with high-quality community and home-based services, including home health agencies. Such partnerships would create opportunities for improved transmission of clinical information between the home and physicians and ultimately improve care for patients. Better data integration would enable hospital and outpatient personnel to use Outcomes Assessment Information Data Set data to track patient status over time. Finally, policies need to support interoperability and collection of standardized data sets across settings, such as the United States Core Data for Interoperability.^10^ Incentives such as the bundled payment programs with financial responsibility and risk sharing 90 days postdischarge are facilitators for collaboration, data sharing, mutual learning, and continuous improvement, with resulting benefits to be experienced by patients (and caregivers) who experience heart failure, as well as by those individuals and organizations that provide their care.

## Funding support and author disclosures

This paper was supported with funding from the 10.13039/100000050National Heart, Lung, and Blood Institute from the National Institute of Health (R01HL169312). The authors have reported that they have no relationships relevant to the contents of this paper to disclose.
